# Methylome profiling of young adults with depression supports a link with immune response and psoriasis

**DOI:** 10.1186/s13148-020-00877-7

**Published:** 2020-07-02

**Authors:** Coral R. Lapsley, Rachelle Irwin, Margaret McLafferty, Sara Jayne Thursby, Siobhan M. O’Neill, Anthony J. Bjourson, Colum P. Walsh, Elaine K. Murray

**Affiliations:** 1 Northern Ireland Centre for Stratified Medicine, School of Biomedical Sciences, Ulster University, C-TRIC, Altnagelvin Hospital, Derry/Londonderry, UK; 2 Genomics Medicine Research Group, School of Biomedical Sciences, Ulster University, Coleraine Campus, Coleraine, UK; 3 School of Psychology, Ulster University, Coleraine Campus, Coleraine, UK

**Keywords:** Depression, Suicide, DNA methylation, Inflammation, Epidermal differentiation complex, Psoriasis

## Abstract

**Background:**

Currently the leading cause of global disability, clinical depression is a heterogeneous condition characterised by low mood, anhedonia and cognitive impairments. Its growing incidence among young people, often co-occurring with self-harm, is of particular concern. We recently reported very high rates of depression among first year university students in Northern Ireland, with over 25% meeting the clinical criteria, based on DSM IV. However, the causes of depression in such groups remain unclear, and diagnosis is hampered by a lack of biological markers. The aim of this exploratory study was to examine DNA methylation patterns in saliva samples from individuals with a history of depression and matched healthy controls.

**Results:**

From our student subjects who showed evidence of a total lifetime major depressive event (MDE, *n* = 186) we identified a small but distinct subgroup (*n* = 30) with higher risk scores on the basis of co-occurrence of self-harm and attempted suicide. Factors conferring elevated risk included being female or non-heterosexual, and intrinsic factors such as emotional suppression and impulsiveness. Saliva samples were collected and a closely matched set of high-risk cases (*n* = 16) and healthy controls (*n* = 16) similar in age, gender and smoking status were compared. These showed substantial differences in DNA methylation marks across the genome, specifically in the late cornified envelope (LCE) gene cluster. Gene ontology analysis showed highly significant enrichment for immune response, and in particular genes associated with the inflammatory skin condition psoriasis, which we confirmed using a second bioinformatics approach. We then verified methylation gains at the *LCE* gene cluster at the epidermal differentiation complex and at *MIR4520A/B* in our cases in the laboratory, using pyrosequencing. Additionally, we found loss of methylation at the *PSORSC13* locus on chromosome 6 by array and pyrosequencing, validating recent findings in brain tissue from people who had died by suicide. Finally, we could show that similar changes in immune gene methylation preceded the onset of depression in an independent cohort of adolescent females.

**Conclusions:**

Our data suggests an immune component to the aetiology of depression in at least a small subgroup of cases, consistent with the accumulating evidence supporting a relationship between inflammation and depression. Additionally, DNA methylation changes at key loci, detected in saliva, may represent a valuable tool for identifying at-risk subjects.

## Background

Depression is a highly prevalent, complex mental health disorder characterised by a range of debilitating symptoms. It affects over 300 million people globally [1] and is responsible for more years lost to disability (YLD) than any other condition, with a total of 76.4 million YLD [2]. Mental health problems, including depression, often emerge before age 18 with the period from 18 to 25 having been highlighted as a susceptible time in a person’s life [3]. In particular, high prevalence rates of mental health problems and suicidality have been found among university students [4, 5]. Northern Ireland (NI) has one of the highest incidences of mental illness in Western Europe [6] and the highest rate of suicide in the UK, a rate which continues to increase [7]. The trans-generational impact of the years of conflict in NI have been mooted as one potential contributor to this [8]. We recently reported on prevalence rates of mental health disorders, self-harm and suicidality in a large cohort (*n* = 739) of first year NI university students [9] and found that, consistent with the other recent studies [10–13], rates were high, with more than 50% of new undergraduate students reporting any lifetime mental disorder. Rates of depression and suicidal ideation were particularly high (24.2% and 31.0% respectively). Consistent with other studies suggesting that self-harm is the strongest predictor of suicidal behaviour [14–17], 122/155 individuals who self-harmed (78.7%) reported suicidal ideation in our cohort [7]. These results highlighted the high incidence of co-occurring depression, self-harm and suicide amongst young people entering university in our study population.

The aetiology of depression is very complex, but epidemiological studies indicate that genetic and environmental interactions are both implicated in disease pathology [18–20]. There is a genetic component to the aetiology of psychiatric disorders, including depression, which has been demonstrated in twin and family studies [21] indicating up to 40% heritability. The most recent meta-analysis of genome-wide association studies (GWAS), including 246,363 cases of depression and 561,190 controls, identified 102 independent regions reaching genome-wide significance associated with depression, including genes and pathways involved in synaptic structure and neurotransmission [22]. In terms of environmental causes, severe childhood adversity and trauma including both verbal and physical abuse, neglect and parental mental disorders are also major contributing factors to the development of mood disorders and suicidal behaviours [23–25]. Other childhood adversities of varying severity, e.g. parental loss, bullying and socioeconomic status are all associated with increased incidence of depression in later life [24, 26].

There are several well-discussed theories of depression including the monoamine theory [27], HPA axis dysregulation in response to stress [28], and in particular the emerging role of inflammation [29, 30]. Epidemiological research indicates that up to 70% of individuals with autoimmune and inflammatory diseases, such as rheumatoid arthritis and heart disease, experience depression [31]. Chronic stress, a major risk factor for depression, can activate inflammatory response in both the periphery and CNS through the hypothalamic pituitary adrenal (HPA) axis [32]. Impaired negative feedback in the HPA axis resulting in high levels of cortisol lead to the production of pro-inflammatory cytokines, chemokines and acute phase proteins from macrophages through the activation of NF-kB [33, 34]. Peripheral inflammatory signals are detected by microglia in the brain, which then initiate their own inflammatory cascade through the activation of CNS cytokines, reactive oxygen species (ROS) and reactive nitrogen species (RNS) [35], ultimately leading to alterations in serotonin signalling and changes in mood.

Peripheral levels of pro-inflammatory cytokines IL-6 and TNF-α are elevated in depression patients who were SSRI resistant compared to patients with depression, but in remission whose cytokine levels were similar to matched healthy controls [36, 37]. In addition, IL-12 and IL-4 were found to decrease in patients receiving a course of sertraline treatment [38]. C-reactive protein (CRP) is elevated in peripheral blood from depressed patients and significantly decreased from baseline following successful treatment with the SSRI, sertraline [37], further support for the link between inflammation and depression and the potential use of immune markers to stratify patients.

Our understanding of these mechanisms on a molecular level however remains poor. The genes implicated in each of these are different. For example, polymorphisms in components of the serotonin system such as the serotonin-transporter-linked *5-HTTLPR* involved with monoamine levels affect predisposition to anxiety and depression [39, 40]. In contrast, polymorphisms in stress-related genes such as 5-HT transporter and *CRF* may instead modify susceptibility to depression according to HPA axis models. More recently, there has been great interest in possible epigenetic rather than genetic changes to components in either the HPA axis, such as glucocorticoid receptor (GR), or the inflammation pathway.

Epigenetic modifications such as DNA methylation, in contrast to DNA polymorphisms, can be influenced by environmental factors and provides a potential mechanism through which life events such as childhood trauma and stress, major risk factors of depression, can lead to the biological, and ultimately behavioural, changes associated with depression [41]. Epigenetic mechanisms could therefore be a key mediator of the interplay between biological vulnerability and life events leading to the behavioural changes seen in depression. In this context, there has been much recent interest in methylation changes at the glucocorticoid receptor (GR) and at genes related to corticotropin releasing hormone action, all with potential roles of an HPA axis model [42–44]. In contrast, a recent study from Murphy and colleagues using the Illumina 450K Beadarray chip uncovered instead significant association between self-reported depression and methylation changes at genes related to immune function in peripheral blood samples, particularly the *LTB4R* and *TRIM39-RPP21* loci, [45]. An earlier study by the same team found significant methylation changes at the *PSORC13* gene, involved in the inflammatory skin condition psoriasis, in completed suicide cases [46]. Given the differing targets implicated in these studies, further work in additional cohorts could help to clarify the main pathways showing epigenetic changes and afford greater insight into potential mechanisms involved.

As indicated above, we reported high rates of depression as well as co-occurring self-harm and suicide risk in a cohort of university entrants [7, 9]. In this study, we wished to (1) investigate the potential external and internal drivers of depression with and without experience of self-harm and a suicide attempt in this cohort; (2) conduct and initial genome-wide screen for DNA methylation differences in a subset of cases with highest levels of risk; (3) verify methylation changes at top-ranking loci using a second method and (4) compare our findings to other recent work in the area.

We were able to confirm that a set of shared risk factors greatly increased the chances of co-occurring depression, self-harm and suicidal ideation. In an initial comparison of saliva samples from students displaying all three conditions and a closely-matched set of controls, we identified significant enrichment for immune response genes among those showing differential methylation. Closer examination highlighted genes involved in psoriasis, including several novel targets (*LCE* and *MIR4520A/B*). All regions could be verified by pyrosequencing. With due consideration of the limitations of the study, these findings nevertheless suggest a significant link between psoriasis and depression, self-harm and suicidal risk that can be detected in peripheral tissues.

## Results

### Risk factors in the student population

In order to determine associations between socio-demographic variables and depression, suicidality and self-harm, logistic regression analysis was undertaken (Table 1). Of the total N=739 students who completed the survey, 24.2% (*n* = 186) showed evidence of a lifetime Major Depressive Event (MDE). We attempted to identify discrete groups within these MDE sufferers using stratification on the basis of risk factors. Several demographic risk factors were significantly correlated with depression, self-harm or suicide attempt, or a combination of these three. In particular, females were more likely to develop depression with comorbid suicide attempt and self-harm *(OR = 3.082*, *p < 0.05*), in comparison with males. Older students (>21 years old) were nearly twice as likely to have depression *(OR = 1.921*, *p < .05)*, compared to students under the age of 21. In contrast to heterosexual students, those who stated they were non-heterosexual were nearly four times more likely to have experienced depression with comorbid suicide attempt and self-harm *(OR = 3.384*, *p < 0.05*). Interestingly, none of the extrinsic factors examined including finances, bullying or maltreatment, were significantly correlated with depression, self-harm and/or suicidality. However, in relation to intrinsic factors such as emotional regulation, students who indicated suppression were more likely to have depression with co-occurring suicide attempt and self-harm (*OR = 1.128*, *p < 0.01*), compared to those who reported reappraisal, which was a protective factor *(OR = 0.924*, *p < 0.01*).

**Table 1:** Logistic regression analyses of correlates of depression

Demographics	Depression only (no SH/attempt)	Dep and self-harm and attempt	Dep and self-harm with no attempt	Dep and attempt with no self-harm
*N* = 739	(*n* = 92)	(*n* = 30)	(*n* = 51)	(*n* = 13)
Demographic risk factors				
Gender				
Female	1.127	3.082*	1.635	1.188
Male	1.0	1.0	1.0	1.0
Age				
21 and over	1.921*	1.161	0.796	3.996
Under 21	1.0	1.0	1.0	1.0
Sexuality				
Non-heterosexual	1.164	3.384*	1.076	1.872
Heterosexual	1.0	1.0	1.0	1.0
Extrinsic risk factors				
Finances				
Enough	0.638	0.939	1.014	1.363
Comfortable	0.816	0.540	0.618	0.000
Well to do	0.308	1.049	1.024	4.438
Poor	1.0	1.0	1.0	1.0
Bullying				
Physical bullying	1.023	1.172	0.825	1.454
Verbal bullying	1.134	1.169	1.319	1.037
Ignoring bullying	1.100	1.033	1.409	1.000
Cyber bullying	0.811	0.961	0.983	1.253
Maltreatment				
Physical Abuse	0.642	0.879	0.883	0.394
Emotional Abuse	1.334	1.540	1.195	1.588
Intrinsic risk factors				
Impulsivity	1.111	1.659**	1.129	0.892
Emotion regulation				
Reappraisal	1.010	0.924**	0.973	0.986
Suppression	1.074**	1.128**	1.016	1.043

All values represent odds ratio; *SH* self-harm; significant results in bold; *p < 0.05, **p < 0.01

### Selection of cases and controls

The logistic regression results suggested a particularly high-risk subpopulation within our study, namely students reporting depression, self-harm and suicidal ideation, which might display epigenetic differences from healthy controls. Of the 739 fully completed Student Wellbeing survey responses a total of only 30 participants reported depression, self-harm and a suicide attempt. As age, gender and smoking status are known confounders in DNA methylation analyses [47, 48], we chose 16 cases for which we could identify closely matched controls based on these criteria, where controls had no life-time history of mental health problems (Table 2). The average age in both groups was 23 years (± 5.4), and they contained equal numbers of males (4) and females (12) each, as well as an identical spread of smoking status (Table 2). All cases had also experienced MDE in the 12 months prior to the survey. The average age of onset of depression was 14, average age of suicide attempt and onset of self-harm was 16 years. On average, cases had experienced a MDE for at least two weeks for an average of 7 years since onset. Saliva samples from these participants were collected and DNA isolated from the samples using standard protocols as described under methods below. Following quality control checks on the DNA, it was then subjected to genome-wide methylation analysis using the Infinium Methylation EPIC 850K Beadchip array.

**Table 2:** Characteristics of samples analysed by EPIC array

Demographics	Controls (*n* = 16)	Cases (*n* = 16)
Age, mean (range ± SD)	23 (18–32 ± 5.4)	23 (18–32 ± 5.0)
Gender		
Male (%)	4 (25)	4 (25)
Female (%)	12 (75)	12 (75)
Smoking status		
Past (%)	1 (6.2)	1 (6.2)
Daily (%)	6 (37.6)	6 (37.6)
Occasional (%)	1 (6.2)	1 (6.2)
Never (%)	8 (50)	8 (50)
Physical health		
Infectious (%)	0 (0)	0 (0)
Blood or immune (%)	0 (0)	1 (6.2)
Endocrine (%)	0 (0)	0 (0)
Eye or ear (%)	1 (6.2)	0 (0)
Neurological (%)	0 (0)	0 (0)
Heart or circulatory (%)	0 (0)	0 (0)
Respiratory (%)	0 (0)	2 (12.5)
Digestive (%)	0 (0)	2 (12.5)
Skin (%)	0 (0)	5 (31.3)
Musculoskeletal (%)	0 (0)	2 (12.5)

### Gains in methylation at immune response genes

**Fig. 1:**
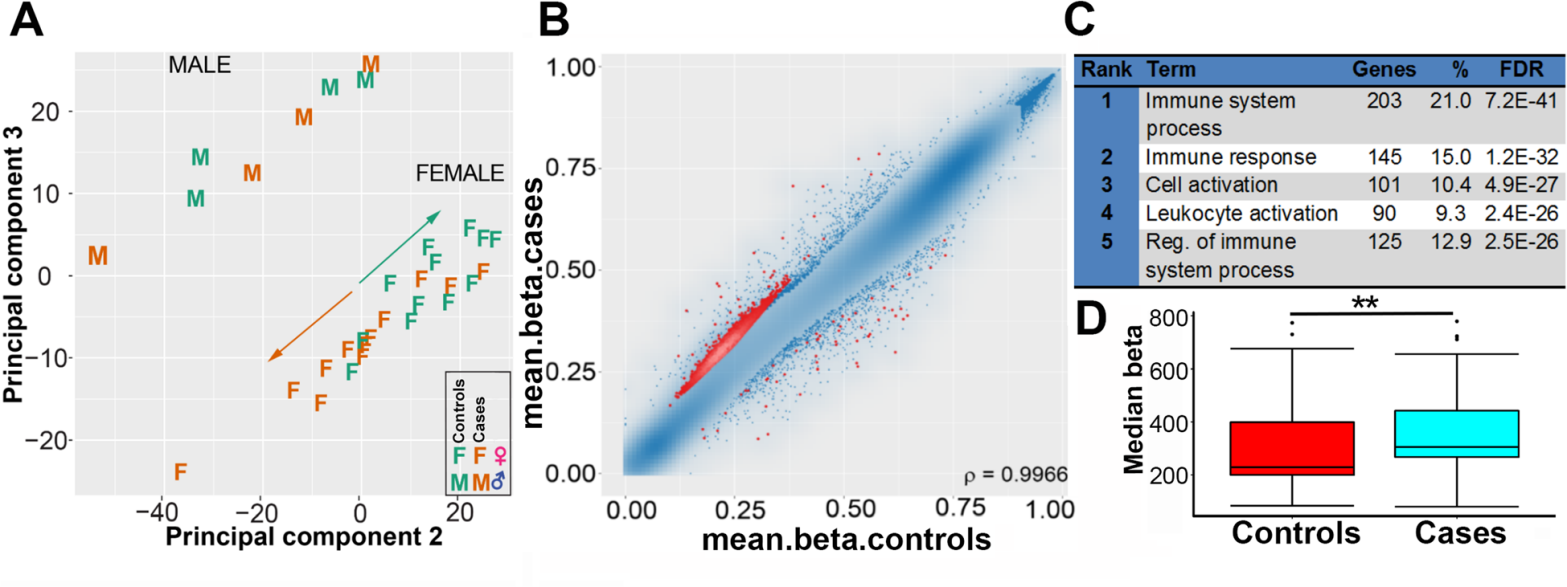
Methylation gains at immune response genes in stratified cases. **a** Principal component analysis (PCA) for all methylated sites of the 32 samples from subjects showing comorbid depression, self-harm and suicidal attempt compared to healthy controls. This indicated clear separation by gender, but also between cases (orange) and controls (green) for females. **b** Scatterplot of differential methylation, with the top 1000 probes in red. **c** Gene ontology analysis of the top differentially methylated gene bodies shows significant enrichment for immune system response terms. **d** Reanalysis of array data using an in-house Galaxy workflow. Differentially methylated probes whose coordinates matched genes from the top two ranks in **c** (relating to immune system response) were identified. Median methylation levels were assessed and again showed a significant difference between cases and controls (**p < 0.01)

Principal component analysis of the methylation status of all 32 samples using the RnBeads analysis package [49] in RStudio demonstrated separation by gender which confirmed established sex differences in methylation (Fig. 1a), but not by smoking status or age (not shown), which indicated that these latter are not major confounding factors in this study. As a control, a quantile-quantile plot was carried out, which showed no evidence of stratification effects among the samples (Suppl. Fig.1). Among the female participants, there was also reasonable separation between cases and controls (Fig. 1a), with female cases clustered in the negative quartiles of the PCA and female controls towards the positive quartiles.

Given that females are at higher risk of co-occurring depression, self-harm and suicide attempt (Table 1) and the separation of cases and controls among females in the PCA (Fig. 1a), we concentrated further bioinformatics analysis on female samples. RnBeads used a combination of the difference in mean methylation (beta value), the quotient of mean methylation and the *p* value to rank the sites showing differential methylation, which we and others have found to be a more reliable indicator of biologically significant differences than *p* value alone, which often highlighted sites showing very small differences in methylation unlikely to be of functional significance. A scatterplot of the top 1000 ranked CpG sites with differential methylation between cases and controls in females displayed predominantly gains of methylation in the cases sample group (Fig. 1b).

In order to determine common features between the top ranking differentially methylated sites, gene ontology (GO) analysis for genes gaining methylation was carried out using DAVID software [50] which indicated strong enrichment scores for immune response terms (Fig. 1c). Common GO term for both promoters and genes included immune system process (GO:0002376), immune response (GO:0006955), cell activation (GO:0001775) and regulation of immune system process (GO:0002682), with very low predicted false discovery rates (FDR). Promoters and genes which showed loss in methylation were instead enriched for GO categories related to epidermal and keratin genes, but with higher FDR rates indicating lower likelihood of being true hits, most likely due to the smaller number of genes showing loss (data not shown).

To verify gains in methylation using a different bioinformatic approach, gene names common across the GO categories related to immune response were compiled (Table S1). We used an in-house developed workflow in Galaxy termed *CandiMeth* (Thursby and Walsh, in prep) to map differentially methylated probes to the human genome map, as previously described [51], found those which fell within promoters (defined as starting 500 bp 5’ of the first exon) of these immune genes, and extracted the mean methylation values in cases and controls. Comparison of the average methylation across these genes between the two groups confirmed significant (*p* < 0.01) gains in mean methylation levels in the cases relative to the controls (Fig. 1d).

### Top-ranking regions include several loci linked with psoriasis and skin conditions

**Fig. 2:**
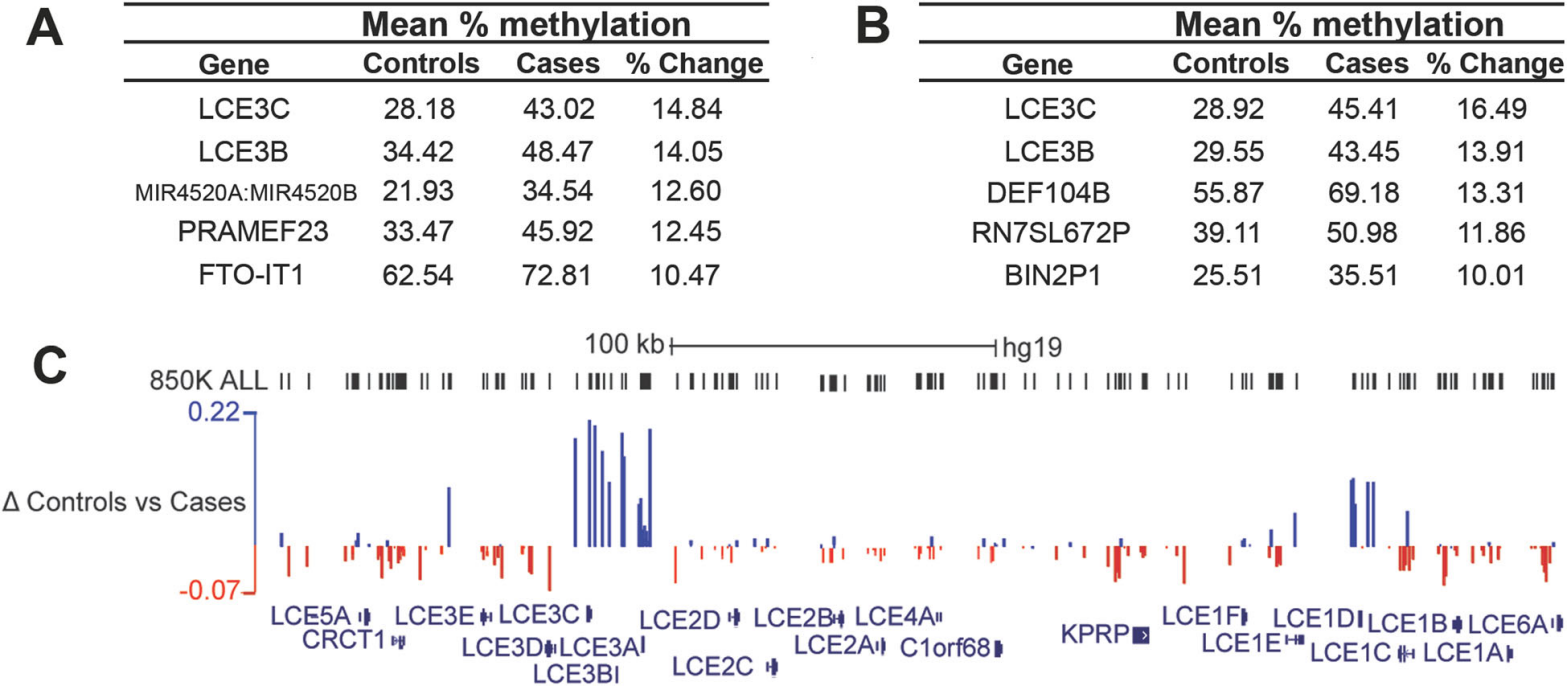
Top-ranking regions are linked to the immune condition psoriasis. **a** The top gene body regions identified from the differential methylation analysis listed by percentage change in methylation (% change). Absolute levels of methylation in % for cases and controls are also shown. The associated gene names from the array manifest file are listed at left and include three with links to psoriasis (*LCE3C*, *LCE3B*, *MIR4520A/B*). **b** Promoters with the largest delta beta gain in methylation levels: *DEFB104B* is also linked to psoriasis **c** Map of part of the Epidermal Differentiation Complex on chromosome 1 from UCSC (GRCh37/hg19 human assembly) showing some of the *LCE* gene cluster (RefSeq track at bottom). The track at top shows the locations of probes from the EPIC array in black (EPIC probes). Below, sites showing differential methylation between cases and controls have been mapped: sites gaining are above the line (blue) and losing below the line (red), with size proportional to change. A scale with maximum and minimum change (Δ) is shown at left. Clusters of probes gaining methylation are seen across *LCE3A-3C* (left) and at *LCE1D* (right). A scale bar is shown at top; kb, kilobase

To examine more closely the immune response targets identified by the genome-wide scan, and to identify those where methylation differences could be verified in the laboratory, we ranked the top hits showing gains in methylation by the magnitude of the difference in methylation (Δβ), both at gene bodies (Fig. 2a) and promoters (Fig. 2b). The Late Cornified Envelope-3C (LCE3C) and -3B (LCE3B) loci featured at the top of both lists and showed substantial gains in methylation (> 10%) in cases versus controls (Fig. 2a, b). These genes are part of a family which encode components of the stratum corneum of the skin and are thought to play a role in skin differentiation. The *LCE*3 genes in particular have been linked to the development of psoriasis, a chronic inflammatory skin disease characterised by hyperproliferation of the epidermis and changes to keratinocyte differentiation [52]. Many of the *LCE* family members are clustered together on chromosome 1q21.3 in a region known as the epidermal differentiation complex (EDC) which contains multiple other genes expressed in the upper layers of the skin. A small deletion encompassing *LCE3B* and part of *LCE*3C (*LCE*3C_*LCE3B*-del) is found in a substantial fraction of psoriasis sufferers [52–54]. As hemizygosity would affect methylation ratios, we checked for copy number variation (CNV) at this locus in our participants using the *R* package *DNAcopy.* No evidence for a CNV on chromosome 1q21 was found using this method, despite successfully detecting an isolated CNV in one patient on chromosome 5 (Fig.S2).

**Fig. 3:**
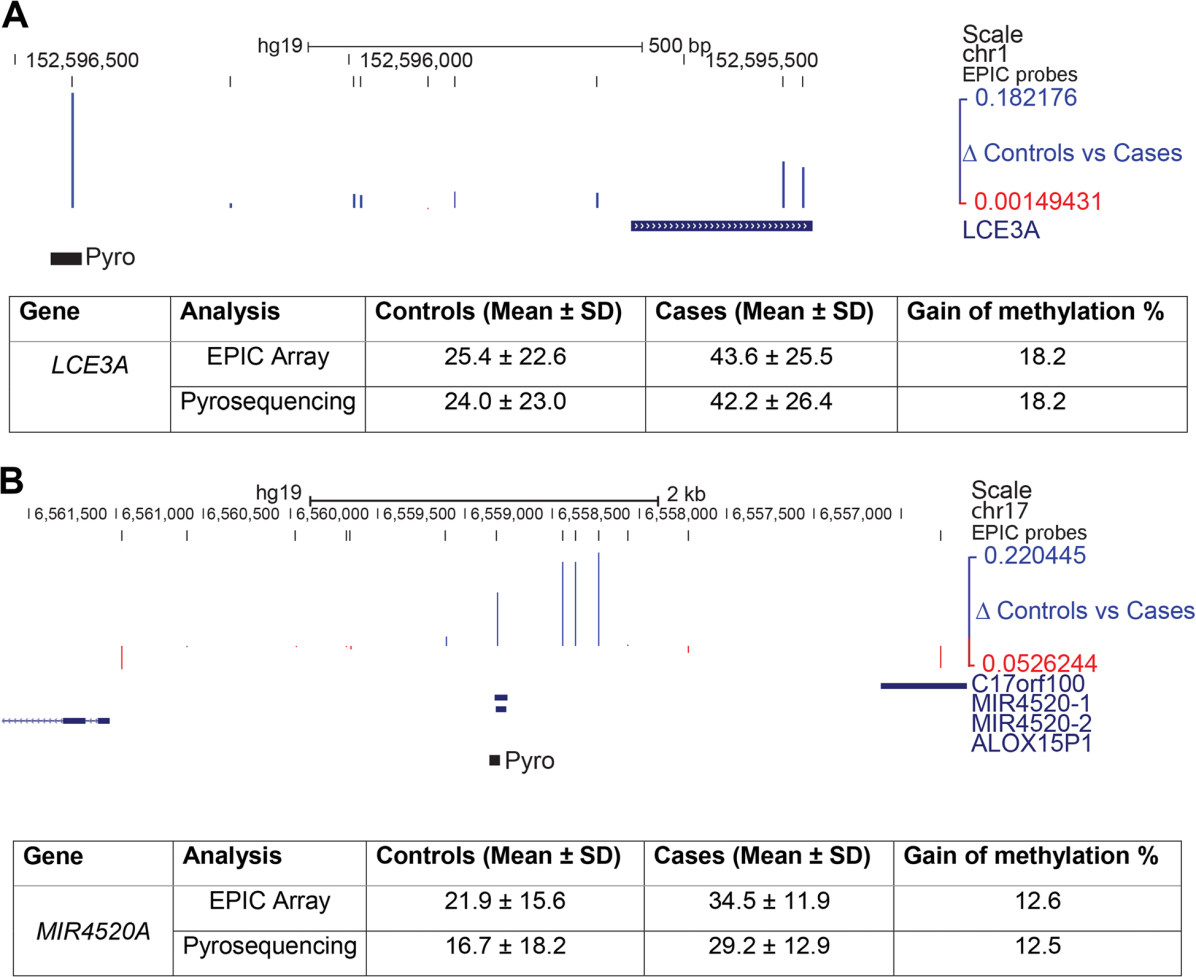
Verification of methylation differences at psoriasis targets. **a** Top: genomic map indicating the region around the *LCE3A* gene. Tracks showing locations of array probes and probes showing differential methylation are as in Fig. 2. The location of the pyrosequencing assay (Pyro) upstream of the first exon of *LCE3A* is also shown. Genomic coordinates are as indicated. Bottom: Table comparing methylation levels as % at the CpG site covered both by an EPIC array probe and the pyroassay, which showed good agreement. **b** Top: genomic map showing the region on chr17 containing the *MIR4520A/B* locus (aka RefSeq *MIR4520-1* and *-2*) and neighbouring genes. Tracks and key are as in a above. Bottom: Table comparing methylation at the CpG covered both by the array and the pyroassay, which were again concordant

We mapped the differentially methylated sites identified in the screen to the human genome (hg19) and found clusters of sites comprising a differentially methylated region (DMR) not only at the *LCE3A-3B* locus, but also further along in the cluster at the *LCE1D-1C* locus (Fig. 2c), further supporting the association of depression with methylation changes at the *LCE* genes in this region. In order to confirm methylation differences at promoter regions, we used a commercially-available pyrosequencing assay to assess a site ~ 700 bp upstream of the *LCE*3A transcriptional start site (Fig. 3a). This site showed a gain of methylation of 18.2% in cases compared to controls (42.2 vs 24.0), identical to that seen using the array (18.2% gain: 43.6% vs 25.4%).

Interestingly another locus the microRNA cluster *MIR4520A/B* on chromosome 17 (Fig. 2a), which was identified as a top hit by the genome-wide assay, has also been linked to psoriasis [55]. We also used a pyrosequencing assay (Fig. 3b) to assess the methylation difference at this region. While absolute levels of methylation at this site were lower by pyroassay than seen using the array (controls 16.7% pyro vs 21.95 array; cases 29.2% vs 34.5%), the approximate level of methylation is similar, and the gains in methylation seen between controls and cases was almost identical (12.5% pyroassay vs 12.6% array) (Fig. 3b).

### Validation of the *PSORS1C3* DMR

**Fig. 4:**
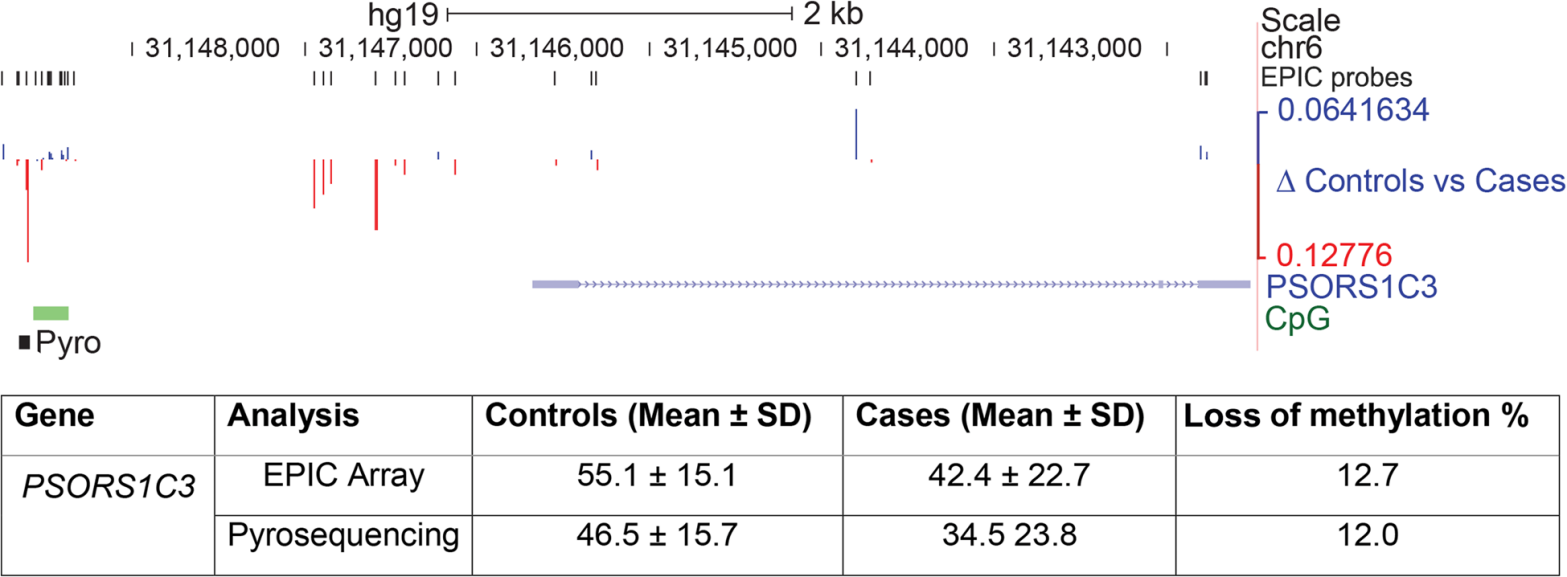
Replication of differential methylation at *PSORSC13* in this cohort. Top: region around the *PSCORSC13* gene, first identified as showing loss of methylation in a separate cohort of depressed subjects who had died by suicide. Symbols are as in Fig. 2 above, with addition of a track for CpG islands (green) often found near promoters. A cluster of probes showing loss of methylation (red, below line) are also evident in our cohort upstream of the first non-coding exon. A pyroassay (pyro, black square) was designed to determine methylation at the individual CpG just 5’ of the island showing greatest loss on the array. Bottom: Table comparing loss of methylation at the indicated CpG site on the array and by pyroassay, which were again in close agreement

Recently an independent epigenetic screen by Murphy and colleagues (2017) also identified a link between psoriasis and depression. On comparing methylation in brain regions BA11 and BA25 of depression-suicide cases and normal controls, they identified Psoriasis Susceptibility 1 Candidate 3 (*PSORS1C3*) as one of the top hits [46]. A region comprising 12 CpG sites across the *PSORS1C3* locus showed loss of methylation (rather than gain) in depression-suicide cases compared to controls in that study. While this locus was not identified as a major target in our screen, there were a number of differences in sample size, tissue type and clinical diagnostics used which could account for this. An examination of the differentially methylated sites in our study confirmed however that there were a number of CpG showing loss of methylation immediately upstream of the *PSORSC1C3* gene in an area likely to contain the promoter (Fig. 4a), with a maximum loss seen of 12.7%. To verify that there were differences in methylation between our cases and controls at this region, we designed a pyroassay for this site, which confirmed a loss of methylation. The results again showed good concordance both in direction (loss) and magnitude (12.0% vs 12.7%) between the pyroassay and the array (Fig. 4b).

### Alterations to immune genes precede development of depression in an independent cohort

**Fig. 5:**
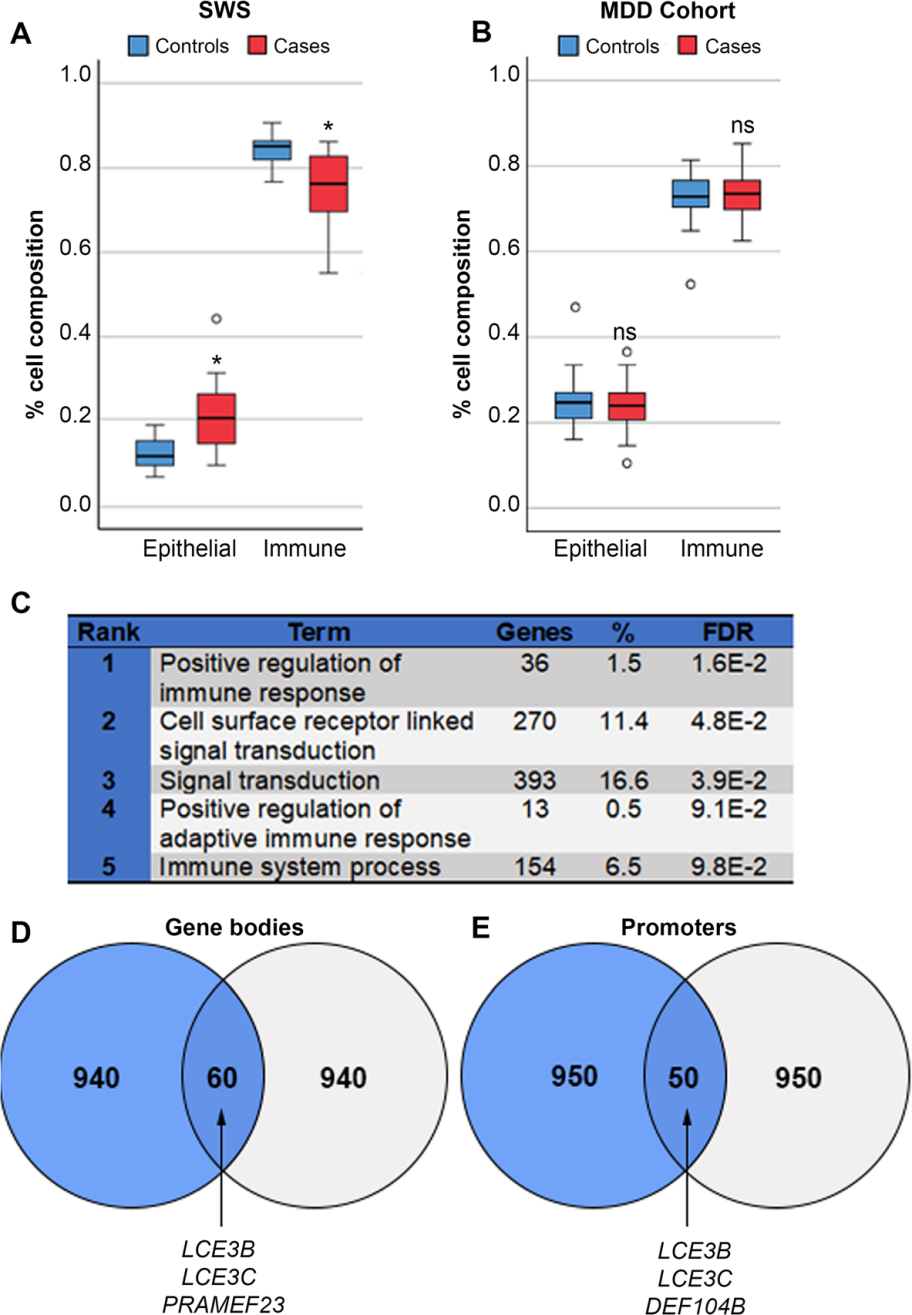
Alterations to immune genes precede development of depression in an independent cohort. **a** EpiDISH cell-type fraction estimation for epithelial and immune cells for the Student Wellbeing Study (SWS); differences were significant by Kruskal-Wallis test (*p < 0.05). **b** EpiDISH analysis of saliva samples taken from at-risk adolescent girls prior to development of major depressive disorder (MDD; 58); ns, not significant, KW test. **c** Gene ontology analysis of the top 3000 ranked differentially methylated gene bodies from the MDD study shows significant enrichment for immune system response terms. **d** Top 1000 gene bodies gaining methylation in the SWS and the MDD cohorts showing overlap; top hits already identified in the SWS cohort are indicated. **e** Top 1000 promoter regions indicating shared targets between the two cohorts as in **d**

One possible complication with regards to working with saliva is that the samples may differ significantly in the ratios of different cell types present. While surrogate variable analysis as performed can compensate for such hidden variables, an estimate of cell counts in the samples would be valuable. However, these could not be done on saliva, so we sought instead to estimate cell ratios using methods based on the array data alone, a so-called reference-free method. These methods are based on the observation that some methylation sites on the array show characteristic levels of methylation in specific tissues, independently of effects at other sites [56]. We employed the recently-developed EpiDISH algorithm [57] which is more suitable for saliva than the original methods developed for blood. As can be seen in Fig. 5a, EpiDISH indicated that the saliva samples from the Student Wellbeing Study cases had significantly different proportions of epithelial (*p* = 0.011, Kruskal-Wallis *H* = 6.16) and immune cells (*p* = 0.013, Kruskal-Wallis *H* = 6.453) from the controls. This suggested that some of the immune gene signature seen in our cohort may be due to differences in immune status at the time of sample collection in the cases versus controls. While this finding is valuable in itself as a biomarker, most of the changes seen in overall methylation and in specific classes of genes will be independent of the small number of sites used to identify tissue type. We wished therefore to further investigate if some of these methylation changes in immune genes may precede the overt changes in cell numbers and be linked to earlier stages in the development of depression.

A recently published study examined methylation patterns in female children, born to mothers with major depressive disorder (MDD), who were at increased risk of developing depression [58]. Saliva was collected from these girls ~ 13 years and analysed with the Illumina EPIC chip, then the children followed longitudinally, with many developing MDD. Due to the excellent match with phenotype, gender, tissue and method of assessment this is an ideal cohort for examining changes which may occur prior to the onset of depression. We obtained raw data from the authors and analysed them using the same pipeline described above. Looking at the children who went on to develop MDD, despite the fact that they show no sign of differences in cell counts between cases and controls (Fig. 5b), we found that many of the same top GO categories involved immune system response (Fig. 5c). Likewise, there was overlap between many of the top-ranked gene bodies (Fig. 5d) and promoters (Fig. 5e) showing gain in methylation between the two studies, with many of the best hits (*LCE3B*, *LCE3C*, *PRAMEF23*, *DEF104B*) being common to both, supporting our theory that immune gene alterations may prefigure MDD.

## Discussion

Our previous work identified a high rate of depression among University students in Northern Ireland [9]. Here, we further analysed this cohort and found that while there were several extrinsic or intrinsic risk factors which were significantly correlated with depression on its own, or depression and any one other feature, depressed students with both self-harm and suicide attempt formed a distinct sub-group with higher risk scores. Significant risk factors for this group included being female or non-heterosexual, and having higher impulsivity and emotional suppression with poorer re-appraisal ability. Hypothesizing that this much smaller sub-group might also show distinct epigenetic marks, we looked for differential methylation patterns in saliva samples from this group. Female cases separated from controls and showed overall tendency to gain in methylation. Methylation differences were significantly enriched in immune-related genes, with a number of top hits, including the *LCE*3 genes and *MIR4520A/B*, being linked to the inflammatory skin condition psoriasis. We confirmed methylation differences at several loci by pyrosequencing. Additionally, the psoriasis gene *PSORS1C3*, recently identified as showing altered methylation in post-mortem brain from suicide completers, was also differentially methylated in our saliva samples. Finally, we saw alterations in methylation at some of the same immune-related genes in an independent cohort of teenage girls prior to onset of depression, suggesting these changes are occurring early in the etiology of the disease.

One of our major findings was the clear differential methylation between cases and controls we identified in immune-related genes. Depression has been previously linked with several chronic inflammatory conditions including diabetes, rheumatoid arthritis and cardiovascular disease [31, 59]. Inflammatory cytokines, IL-6, TNF-α and IFN-γ are consistently upregulated in individuals with depression [32, 60] and many antidepressant medications have anti-inflammatory effects [61]. Furthermore, recent methylome analysis of whole blood also reported that depression-related methylation differences were enriched in pathways related to immune function [45], consistent with what we have identified for the first time in saliva. DMRs in immune response genes and their link to immune dysregulation warrant further investigation as potential biomarkers for depression.

Psoriasis is one of the most common inflammatory skin conditions, and affects up to 125 million people worldwide [62]. While being noncontagious and nonlethal, it nevertheless can be painful and disfiguring and can lead to severe disruptions in everyday social interactions and personal relationships. Psoriasis tends to develop between the ages of 15 and 25 and can lead to an impairment of social development due to attendant feelings of self-consciousness and embarrassment [63]. The average age of onset for psoriasis therefore shows notable overlap with that for depression, commonly occurring around 15 years old [3]. In a large UK population-based study individuals with severe psoriasis were also reported to have an increased susceptibility to depression, anxiety and suicidality [64]. In that study, patients with psoriasis had a 39% increased chance of depression, which increased to 72% for severe psoriasis. Significantly, randomized controlled trials have shown that control of psoriasis symptoms can lead to improvements in psychological outcomes [65, 66]. Paediatric patients in particular have been shown to be at increased risk of developing depression [67]. Individuals with psoriasis display fatigue and sleep deprivation, which has been linked to the concomitant pruitis (itching) and pain and is linked to depression and obstructive sleep apnea in this group [68]. Insomnia has been recently highlighted as a particular risk factor for self-harm and suicide in university students [7, 69].

As indicated earlier, genes in the *LCE* cluster, and particularly *LCE*3 homologues, have been strongly linked with psoriasis. The genes lie in the Epidermal Differentiation Complex (EDC) on chromosome 1, and genome-wide association studies (GWAS) have identified a major psoriasis susceptibility locus (*PSORS4*) in this region [70, 71]. A separate GWAS in the Chinese Han population identified two SNPs in the *LCE3A* gene, and three in *LCE3D* as particularly associated with psoriasis. The EDC also contains other skin genes such as *Filaggrin family member 2* (*FLG2*) and *Cornulin*: interestingly experimental disruption of the skin barrier resulted in down-regulation of *LCE5A*, *LCE2B* and *FLG2* but upregulation of *LCE3A*, *Involucrin* and *Hornerin* [72]. *LCE*3 genes show marked difference in expression between psoriatic lesions and normal skin, but not between pre-lesional skin and control [72–74], consistent with roles in skin repair rather than development per se. A small deletion encompassing parts of *LCE3C* and *LCE3B* (*LCE3C_LCE3B*-del) has also been identified as a risk factor for psoriasis in a number of populations [52]. While we tested for CNV affecting this region in our cohort, we found no evidence for any deletions. The *LCE* cluster has also been reported to interact epistatically with the *PSORS1* locus at the HLA-complex on Chromosome 1 [53, 75].

Furthering the link with psoriasis, other top hits from our screen are also connected with this condition. The *MIR4520A/B* locus, which produces two microRNA *miR4520A* and *–B*, was also a top-ranking region from our screen. While little is currently known about this microRNA, next-generation sequencing of small RNAs from normal versus psoriatic skin highlighted *miR4520A* as one of the most abundant novel miRNA expressed in psoriatic skin [55] which was significantly downregulated in psoriatic lesions. Although it has not been firmly established whether transcription of this miR is epigenetically controlled, increased DNA methylation at this region may indicate downregulation of this gene in our cohort. Another top hit was *DEFB104B*, which is a beta-defensin and part of a family of antimicrobial and cytotoxic peptides made by neutrophils. Defensins are expressed during inflammatory conditions, including psoriasis [76]. A total of 4 CpG sites were hypermethylated at the promoter regions of *DEFB104B* in our cases, suggesting perhaps a form of silencing or reduced mRNA expression and thus reduction/suppression of the innate immune response in depressive cases.

As detailed above, a number of loci involved in psoriasis were identified among our cases with depression and co-occurring self-harm and suicide attempt. Recently, Mill and colleagues (2017) compared methylation in two cortical brain regions from depressed suicide completers and non-psychiatric sudden-death controls and also identified a psoriasis susceptibility locus *PSORS1C3* as the main target affected [46]. They found a loss of methylation at a DMR upstream of the gene, which they verified by pyrosequencing and could replicate in a second set of suicide samples. While *PSORSC1C3* was not identified as a high-ranking DMR in our screen, there were a number of important differences between our studies: (1)we were using saliva samples not brain, and tissue type is known to have a major effect on methylation patterns [77] as seen even between brain regions [46]; (2) our samples were from subjects reporting suicide attempt at most, not completion and (3) there were a number of technical differences in diagnosis, processing and analysis, including the use of different chips (450K vs 850K EPIC). Nevertheless, on examination of the *PSORS1C3* locus in our cohort we found a DMR at this region which also showed loss of methylation in our samples (in contrast to the gains seen at other loci). We could also verify this using pyrosequencing, with good agreement in direction and magnitude of difference. These results are important as they (1) confirm methylation changes at this locus in another cohort displaying depression; (2) further link psoriasis, depression and suicidal thoughts and behaviour; (3) indicate that changes seen in the brain may also be mirrored in peripheral tissues such as saliva (4) suggest the change precedes death by suicide and therefore may have utility as a predictive tool.

The exact nature of the overlap between depression and psoriasis warrants further investigation. Traditionally, depression was thought to be a secondary consequence of living with a chronic physical condition such as psoriasis. However, the accumulating evidence that depression itself has an inflammatory component suggests that there may be common aetiology which can lead to mental health disorders, physical health problems, or both in a given individual. In our current sample, none of the depressed group reported psoriasis, indicating that while there is overlap in risk on a molecular level this does not necessarily manifest as co-occurrence of the two conditions.

While replication of the *PSORSC13* finding in saliva is encouraging, there has been debate over whether findings in peripheral tissue in general will parallel those in the organ most likely to be primarily involved, in this case the brain. A recent study evaluated DNA methylation patterns in the blood and saliva using the 450K BeadChip to assess the correlation of the two sample sources with secondary data from brain tissue. Although concordance was poor overall, methylation patterns in saliva were more similar to the brain methylome than blood [78]. The development of biomarkers that can be used to improve the diagnosis of depression, or those predictive of response to treatment, requires them to be easily accessible for sampling, so identification of reliable markers of depression in the periphery have more clinical utility than those in the brain. Saliva is a very promising potential biomarker discovery tissue due to the non-invasive sampling method. A concern is whether cell composition differences between cases and controls might be a confounding effect. The collection method utilised here involved the lysis of the cells so the specific cell types present could not be directly assessed. EpiDISH analysis indicated that the saliva samples from the cases had different proportions of both cell types from the controls, and that these were significant. Thus, it is likely that the methylation profile in part may reflect a difference in cell count in the cases vs controls. From the point of view of a biomarker, this is still a valuable finding as it can help to identify people with depression based on a heightened altered cell profile. On another level, we must consider that the surrogate variable analysis (SVA) and correction applied to our cohort will have accounted in part for this, suggesting that the immune gene hits highlighted in the analysis are genuine targets: these two levels of information are analogous to the cell count estimators such as EpiDISH, which can determine cell types independently of the top hits in the differential methylation analysis. Furthermore, immune genes were the top GO categories, and LCE and other immune genes the top hits, in the independent MDD cohort where there was no evidence of cell count skewing and prior to MDD onset, strongly supporting an underlying immune system link with depression.

The current exploratory study was carried out on a relatively small subgroup from the larger available student cohort as an initial investigation into the viability of using DNA methylation in saliva samples as potential biomarkers of depression. However, we have taken a stratified approach here in sub-classifying the cases of depression and, using logistic regression identified the small group of students who represent the most severe cases of depression with self-harm and suicide attempt. Our sample screened by array here represented more than half (16/30) of that high-risk subgroup: by limiting the samples chosen we could more closely match these cases to controls with no history of mental disorders in terms of known confounders in methylation analysis, namely age, gender and smoking. The stratification approach may explain why clear differences were observed between the groups despite the small sample size overall. Significant hits were determined using a combined rank approach across adjacent sites, which took into account not only *p* value, but also magnitude and quotient for the changes in methylation, and is considered a more reliable indicator of biologically meaningful differences than *p* value alone [79]. Methylomic profiling of additional samples across a broad spectrum of individuals with depression are necessary to determine whether these changes are representative of depressive cohorts generally and to assess their utility as biomarkers.

## Conclusions

While this study is exploratory in nature, and has a number of caveats as indicated above, it nevertheless shows a novel linkage between epigenetic changes detected in saliva, and a particular category of depression with self-harm and suicidal attempt. Furthermore, our study clearly implicates changes at genes involved in the chronic inflammatory condition psoriasis, supporting emerging evidence from a number of epidemiological studies. Future work will include the analysis of a larger cohort if possible, as well as investigating specific intrinsic influences such as childhood adversity, and other clinical/phenotypic information. Additionally, it will be valuable to explore the potential mechanistic role of methylation in controlling transcription from these loci. Further analyses will also determine whether these markers have clinical utility in identifying or sub-classifying depression, or in predicting therapeutic response.

## Methods

### Ethics

Ethical approval was obtained from Ulster University Research Ethics Committee (REC/15/0004).

### Design

The Ulster University Student Wellbeing Study (UUSWS) has been described in detail elsewhere [7, 9] and was conducted as part of the WHO World Mental Health International College Student Project (WMH-ICS). The UUSWS study is being conducted as part of the WHO World Mental Health International College Student Project (WMH-ICS). An observational, longitidunal cohort study design is used for all studies. Prospective studies, such as this, can be very benefical in that recall issues are minimised, sequences or patterns of events can be established and causal relationships may be inferred.

### Recruitment

All students commencing Ulster University in September 2015 were emailed a participant information sheet. First year students were recruited during registration where they gave written consent, provided a saliva sample and were given a unique, anonymous number to complete an online mental health survey clinically validated against the Diagnostic and Statistical Manual of Mental Disorders (DSM-IV) [80].

### Survey responses

The survey instrument was adapted from the WMH Composite International Diagnostic Interview (CIDI), version 3.0 [81], designed to be validated against the criteria of ICD-10 and DSM-IV disorders. Although these measures are self-report, good concordance has been found between the CIDI and clinical assessments [82]. Participants completed a section on emotional problems including depression, bi-polar disorder, anxiety, panic attacks or panic disorder and other serious emotional problems. Suicidal behaviour and non-suicidal self-injury (NSSI) questions were included from the Self-Injurious Thoughts and Behaviours Interview [83]. Impulsivity was measured by asking the participants if they often act without thinking, a Likert scale ranging from 1 ‘strongly agree’ to 6 ‘strongly disagree’ from the Student Experience and Student Expectations questionnaire [84]. Bullying was measured by asking participants how often they experienced the following: (1) you were bullied at school physically (i.e. repeatedly punched, shoved or physically hurt)? (2) You were bullied at school verbally (i.e. teased, called names). (3) You were bullied at school by someone who purposefully ignored you, excluded you, or spread rumours about you behind your back? You were bullied over the internet (e.g. Facebook, Twitter) or by text messaging? The questionaire used a Likert scale ranging from 1 ‘very often’ to 5 ‘never’. These questions were adapted from The Bully Survey [85]. Maltreatment was measured by asking participants how often they experienced the following using a Likert scale ranging from 1 ‘very often’ to 5 ‘never’: (1) physical abuse—you were physically abused at home; (2) emotional abuse—you were emotionally abused at home. The questions were adapted from the Adverse Childhood Experiences Scale [86]. Emotion regulation was measured using the Emotion Regulation Questionnaire, which consists of two dimensions of emotion regulation, reappraisal (six questions) and suppression (four questions), related to how well they control or manage their emotions. The instrument utilises a 7-point Likert scale. High scores for reappraisal are optimal while low scores for suppression indicate better emotion regulation strategies [87]. Logistic regression analysis was used to explore relationships between socio-demographic risk factors for individuals with depression, and comorbid suicidality and/or self-harm.

### Case selection

Cases (*n* = 16) were selected from students who met the criteria for life-time (LT) major depressive episode, and who also reported suicide attempt and self-harm. Life-time depression is determined based on the response to seven questions (Likert scale) corresponding to DSM-IV criteria for depression. To calculate LT depression the first 6 symptoms/questions were recoded to 4 = “all or most of the time and 0 = none of the time, and summed. If at least 1 of the first 4 symptoms was “all or most of the time” and the sum of all six symptoms was at least 15 then participants met the criteria for depression. Suicidality, including thoughts, plans and attempts and self-harm, was assessed using items from the Self-Injurious Thoughts and Behaviour Interview [83]. If a participant responded yes to either of two questions asking about thoughts of hurting or killing themselves, or responded yes to direct questions on plan or attempt, they met the criteria for suicidal behaviour. Self-harm was assessed by asking the participant the following question: did you ever do something to hurt yourself on purpose, without wanting to die? (e.g. cutting yourself, hitting yourself, or burning yourself)? If they responded yes, they met the criteria for self-harm and were asked some further questions with regards to the number of times and what age this began. Healthy controls (*n* = 16) were participants who reported no mental health problems, and strictly matched by age, gender and smoking status.

### Sample collection

Saliva samples were collected using Oragene OG-500 kits (DNA Genotek, Ontario Canada), enabling the self-collection and stabilisation of DNA at room temperature as per manufacturer guidelines.

### DNA extraction, bisulphite conversion and EPIC Beadchip Array

Saliva samples were incubated for 2 h at 56 °C, and DNA isolation carried out with PrepIT.L2P (DNA Genotek Inc., Canada) as per the manufacturer’s instructions. The purity and integrity of the genomic DNA preparations were assessed by agarose gel electrophoresis, and the quantity of DNA was determined using Quant-IT PicoGreen dsDNA Assay Kit (Invitrogen, Paisley, UK). In preparation for DNA methylation analysis, 500 ng of DNA was bisulphite converted using the EZ DNA Methylation Kit (Zymo Research, CA, USA) according to manufacturer’s instructions. Genome-wide DNA methylation profiles were generated using the Infinium Methylation EPIC Beadchip Array, and the Beadchip images captured using an Illumina iScan (Cambridge Genomic Services, Cambridge, UK) for matched cases (*n* = 16) and controls (*n* = 16).

### Bioinformatic analysis

Data was analysed using the *RnBeads* package (version 1.6.1) [49] on the freely available statistical software platform *R* (version 3.1.3). All samples passed quality control and were subjected to pre-processing, which involves filtering of probes and normalisation. Probes removed included those with a missing value (NA), probes at SNP-enriched sites, and bad quality probes determined by *greedycut* algorithm. Background correction was carried out using *methylumi.noob v2.32.0* [88] and the methylation values of the remainder probes were normalized using *bmiq* [89]. Copy number variation (CNV) was assessed using the *DNAcopy* package v1.60.0 [90]. In order to account for any hidden confounding variables in the dataset, surrogate variable analysis was carried out using the *limma* method [91]. The methylation intensities for each probe, representing a CpG site, were represented as β values (ranging from 0, unmethylated, to 1, fully methylated) and these were plotted against genomic loci (based on Human Genome Build 19) using an in-house developed workflow in GALAXY v19.01 (https://usegalaxy.org/) [92] called *CandiMeth* (Thursby and Walsh, in prep) in order to visualise changes in DNA methylation in UCSC (https://genome.ucsc.edu/) and quantify differences across specific genomic intervals. Subsequent gene ontology (GO) analyses were performed using DAVID v6.7 (https://david.ncifcrf.gov/) [50]. Cell type composition estimation was performed in RStudio using EpiDISH v2.2.2 [57].

### Pyrosequencing

Bisulphite pyrosequencing was carried out in order to verify changes in methylation at loci of interest from the Infinium MethylationEPIC Beadchip Array. Primers spanning the probes of interest from the array were designed using the PyroMark Assay Design Software 2.0 (Qiagen, Manchester UK). Bisulfite-treated DNA was PCR-amplified using the PyroMark PCR kit (Qiagen, Hilden, Germany) according to manufacturer’s instruction. The primer sequences and PCR conditions are summarized in Supplementary Table 1. Amplification was carried out as follows: 95 °C for 15 min, followed by 45 cycles of 95 °C for 30 s, 56 °C for 30 s, and 72 °C for 30 s, with a final elongation step at 72 °C for 10 min. Pyrosequencing was performed as per manufacturer’s instructions on the PyroMark Q24 system (Qiagen, Hilden, Germany), and methylation levels were analysed using PyroMark Q24 1.010 software (Qiagen, Hilden, Germany).

### Statistical analysis

Differential methylation analysis was conducted on site and region level for healthy controls and cases samples. The normalized β values of the Infinium MethylationEPIC Beadchip Array data were converted into *M* values (*M* = log2(β/(1-β)) and differential methylation between samples (cases vs. healthy controls) was estimated with hierarchical linear models using limma. On the region level (i.e. genes, promoters, CpG islands), differential methylation was computed based on the average difference in means across all sites in a specified region of the sample groups and the mean of quotients in mean methylation as well as a combined p-value, which was calculated from all site p-values in the region using a generalization of Fisher's method [93]. In addition, each region was assigned a rank based on each of these criteria. The smaller the combined rank for a region, the more evidence for differential methylation it exhibits.

The top 1000 ranking genes of each region was input into DAVID. We used DAVID software to determine significance of each gene ontology category, calculated using a modified Fisher’s exact test (EASE score) which was FDR-corrected. Pyrosequencing data were analysed using Student’s *t* test to identify statistical differences between cases and healthy controls. EpiDISH data was analysed using Kruskal-Wallis test to identify statistical differences between cell type composition for cases and controls in each cohort. A *p* value < 0.05 was considered significant.

## Supplementary information

**Additional file 1: Table S1.** Pyrosequencing primers.

**Additional file 2: Table S2.** Immune-related genes analysed for gains in methylation at promoter.

**Additional file 3: Figure S1.** Absence of population substructure effects. A quantile–quantile (QQ) plot showing observed vs. expected − log10 (*p* values) for association at all CpG sites. The x-axis shows the expected −log10 (*p* value), the y-axis the observed –log10 (*p* value): the red line indicates the expected distributions under the null hypothesis and the black dots were the observed values. A close match at lower significance values indicated no systematic inflation of P was seen due to unaccounted-for stratification effects.

**Additional file 4: Figure S2.** Absence of deletions or duplications at top differentially methylated loci. EPIC array probe data was analysed using the *DNAcopy* package in R to look for variations indicating copy number variation (CNV): an example output plot from subject 225 (Healthy Control) is shown. Probe index number is shown along the x-axis, while gain/loss in copy number, expressed as the log-10 ratio, is shown on the Y-axis; dots coming away from the line indicate probes showing gains or losses of signal consistent with regional duplications/deletions. No significant CNVs were detected in the Epidermal Differentiation Complex (EDC) region on chromosome 1q21, but the approach successfully detected a CNV on chromosome 5 in one participant (arrow at right) not overlapping any of the differentially methylated regions, shown here as a positive control for sensitivity. *DNAcopy* plots were carried out for all samples and failed to detect copy number variation (CNV) at other top hits.

## Data Availability

The datasets used and/or analysed during the current study are available from the corresponding author on reasonable request.

## Abbreviations

MDE: Major Depressive Episode
LCE: Late cornified envelope
MIR4520A/B: MicroRNA 4520 A/B
PSORSC13: Psoriasis Susceptibility 1 Candidate 3
YLD: Years lost to disability
5-HTTLPR: Serotonin-transporter-linked receptor
5-HT: 5-HT transporter
CRF: Corticotropin-releasing factor
GR: Glucocorticoid receptor
LTB4R: Leukotriene B4 Receptor
TRIM39-RPP21: Tripartite motif-containing 39 - Ribonuclease P Protein Subunit P21
SH: Self-harm
CpG: Cytosine guanine dinucleotide
GO: Gene Ontology
EDC: Epidermal differentiation complex
CNV: Copy Number Variation
DMR: Differentially methylated region
IL6: Interleukin 6
TNF-α: Tumour Necrosis Factor alpha
IFN-γ: Interferon Gamma
PSORS4: Psoriasis susceptibility 4
FLG2: Filaggrin family member 2
HLA-complex: The human leukocyte antigen complex
DEFB104B: Defensin Beta 104B
CIDI: Composite International Diagnostic Interview
NSSI: Non-suicidal self-injury
LT: Lifetime
