# Supplementary figures and images for: Methylome profiling of young adults with depression supports a link with immune response and psoriasis
Source: Clin Epigenetics. 2020 Jul 2;12:85. doi: 10.1186/s13148-020-00877-7 (PMC7477873; doi:10.1186/s13148-020-00877-7)

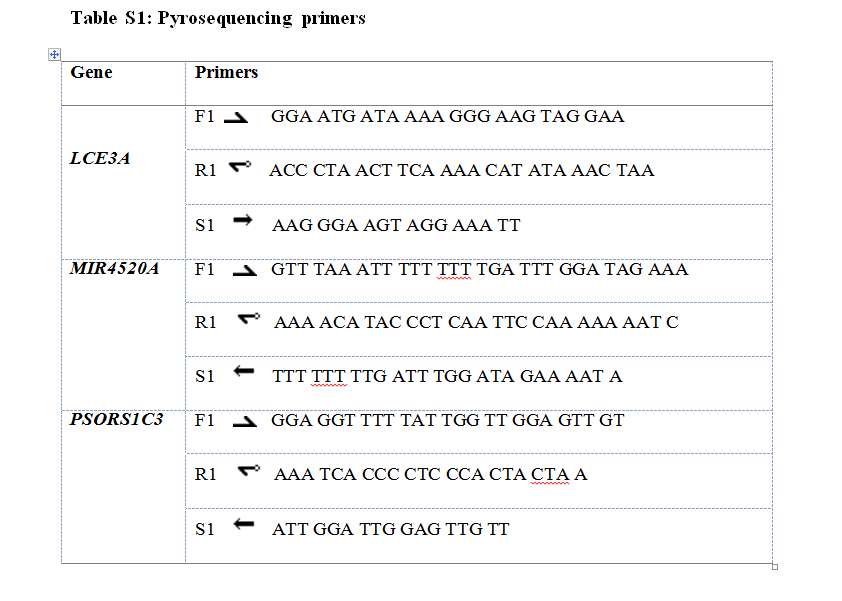

Supplement: Supplementary file 1 — Additional file 1: Table S1. Pyrosequencing primers. [file 13148_2020_877_MOESM1_ESM.png]

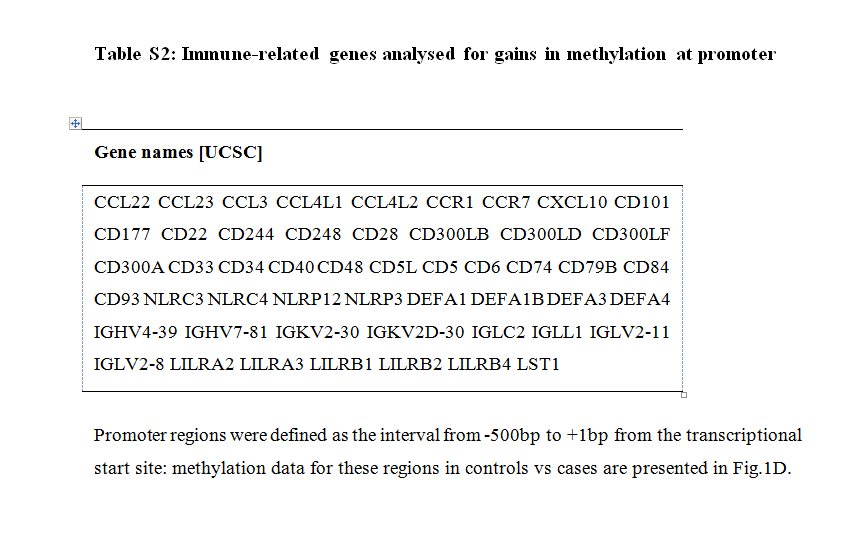

Supplement: Supplementary file 2 — Additional file 2: Table S2. Immune-related genes analysed for gains in methylation at promoter. [file 13148_2020_877_MOESM2_ESM.png]

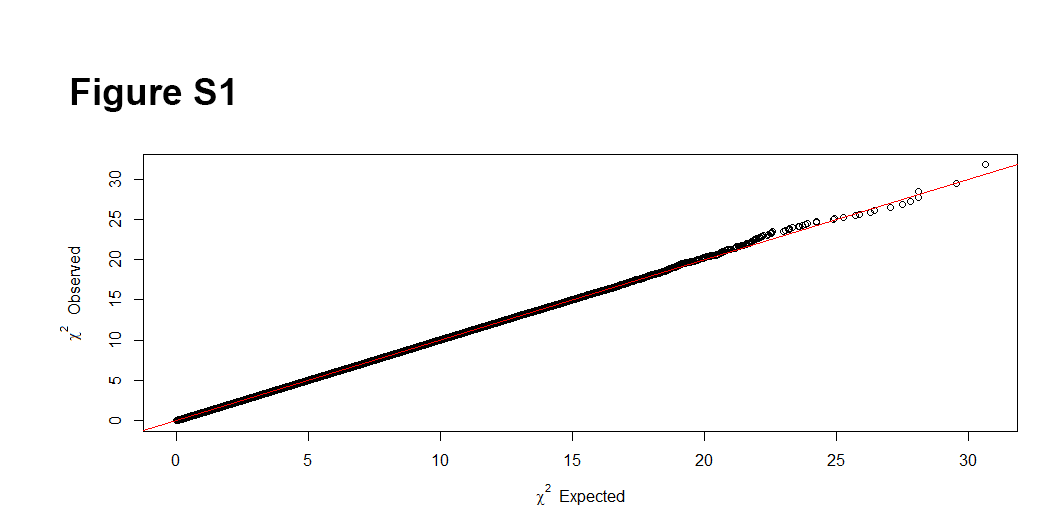

Supplement: Supplementary file 3 — Additional file 3: Figure S1. Absence of population substructure effects. A quantile–quantile (QQ) plot showing observed vs. expected − log10 (p values) for association at all CpG sites. The x-axis shows the expected −log10 (p value), the y-axis the observed –log10 (p value): the red line indicates the expected distributions under the null hypothesis and the black dots were the observed values. A close match at lower significance values indicated no systematic inflation of P was seen due to unaccounted-for stratification effects. [file 13148_2020_877_MOESM3_ESM.tif]

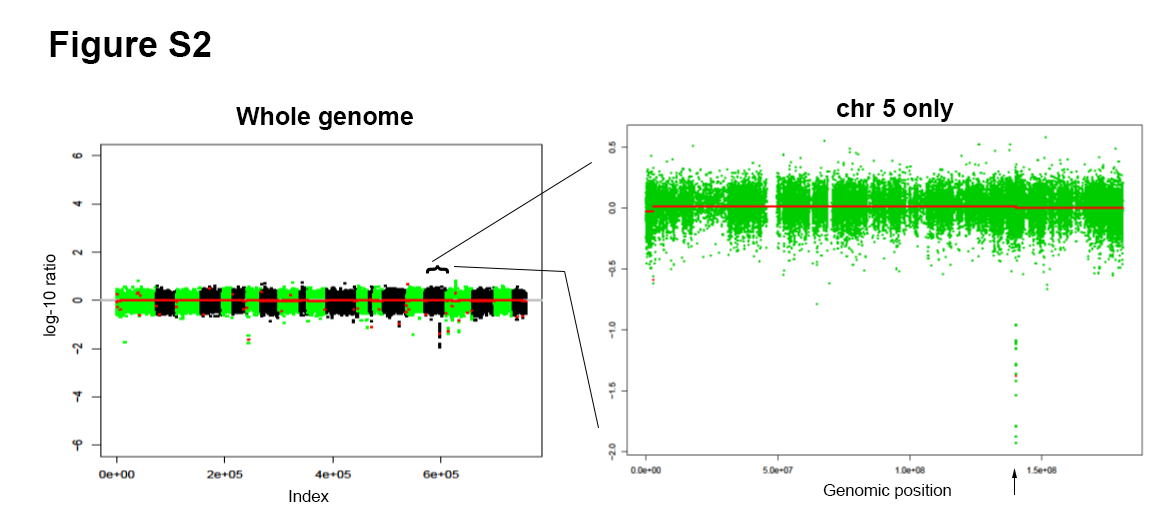

Supplement: Supplementary file 4 — Additional file 4: Figure S2. Absence of deletions or duplications at top differentially methylated loci. EPIC array probe data was analysed using the DNAcopy package in R to look for variations indicating copy number variation (CNV): an example output plot from subject 225 (Healthy Control) is shown. Probe index number is shown along the x-axis, while gain/loss in copy number, expressed as the log-10 ratio, is shown on the Y-axis; dots coming away from the line indicate probes showing gains or losses of signal consistent with regional duplications/deletions. No significant CNVs were detected in the Epidermal Differentiation Complex (EDC) region on chromosome 1q21, but the approach successfully detected a CNV on chromosome 5 in one participant (arrow at right) not overlapping any of the differentially methylated regions, shown here as a positive control for sensitivity. DNAcopy plots were carried out for all samples and failed to detect copy number variation (CNV) at other top hits. [file 13148_2020_877_MOESM4_ESM.tif]
